# Classification of Monkeypox Images Using LIME-Enabled Investigation of Deep Convolutional Neural Network

**DOI:** 10.3390/diagnostics13091639

**Published:** 2023-05-05

**Authors:** M. Lakshmi, Raja Das

**Affiliations:** Department of Mathematics, School of Advanced Sciences, Vellore Institute of Technology (VIT), Vellore 632014, Tamil Nadu, India; llakshmi407@gmail.com

**Keywords:** deep learning, ensemble models, transfer learning, image processing, k-means clustering, LIME, machine learning, support vector machine

## Abstract

In this research, we demonstrate a Deep Convolutional Neural Network-based classification model for the detection of monkeypox. Monkeypox can be difficult to diagnose clinically in its early stages since it resembles both chickenpox and measles in symptoms. The early diagnosis of monkeypox helps doctors cure it more quickly. Therefore, pre-trained models are frequently used in the diagnosis of monkeypox, because the manual analysis of a large number of images is labor-intensive and prone to inaccuracy. Therefore, finding the monkeypox virus requires an automated process. The large layer count of convolutional neural network (CNN) architectures enables them to successfully conceptualize the features on their own, thereby contributing to better performance in image classification. The scientific community has recently articulated significant attention in employing artificial intelligence (AI) to diagnose monkeypox from digital skin images due primarily to AI’s success in COVID-19 identification. The VGG16, VGG19, ResNet50, ResNet101, DenseNet201, and AlexNet models were used in our proposed method to classify patients with monkeypox symptoms with other diseases of a similar kind (chickenpox, measles, and normal). The majority of images in our research are collected from publicly available datasets. This study suggests an adaptive k-means clustering image segmentation technique that delivers precise segmentation results with straightforward operation. Our preliminary computational findings reveal that the proposed model could accurately detect patients with monkeypox. The best overall accuracy achieved by ResNet101 is 94.25%, with an AUC of 98.59%. Additionally, we describe the categorization of our model utilizing feature extraction using Local Interpretable Model-Agnostic Explanations (LIME), which provides a more in-depth understanding of particular properties that distinguish the monkeypox virus.

## 1. Introduction

A viral zoonosis called monkeypox is endemic to some regions of Africa. Its primary symptoms, like those of other illnesses brought on by *pox viruses*, are fever and skin lesions. Unfortunately, a small percentage of individuals may experience severe, multi-systemic illness that quite frequently can be deadly. Due to a rapid outbreak of cases discovered outside of its endemic range, monkeypox has lately attracted attention and worry on a global scale. The smallpox (variola) virus and the monkeypox virus are both enveloped double-stranded DNA viruses belonging to the *Poxviridae* family. In the Democratic Republic of the Congo, monkeypox was first found in a human in 1970 [1]. Several epidemics have occurred since then, primarily impacting African nations. Subsequently, instances outside of Africa were also documented, and the illness gained relevance for public health. The World Health Organization (WHO) has recently confirmed an atypical outbreak of monkeypox from multiple non-endemic countries with increasing numbers of cases reported almost daily. The WHO has declared monkeypox to be a public health emergency of global concern [2]. Monkeypox outbreaks have been documented in 75 nations so far, and they are rapidly expanding around the globe.

Typically, monkeypox is a self-limiting illness with symptoms that last between two and four weeks. Children are more likely to experience severe symptoms, which are connected to the level of viral exposure, the patient’s condition, and the kind of problems. The results might be worse if immunological deficits are present. Although smallpox immunization has proved to be protective in the past, people in the age group of 40 to 50 may now be more vulnerable to monkeypox due to the worldwide discontinuation of smallpox vaccine programs after the illness was eradicated. Monkeypox complications can include secondary infections, bronchopneumonia, sepsis, encephalitis, and corneal infections with subsequent vision loss. It is uncertain how widespread an asymptomatic infection could be.

Whereas this skin lesions and rashes of monkeypox frequently resemble those of other poxes, such as chickenpox and cowpox, its clinical characteristics match those of smallpox. Furthermore, due to its resemblance to measles and chickenpox, it is difficult to detect at an early stage. Because of these parallels, it might be difficult for some medical practitioners to diagnose monkeypox simply by looking at the visual characteristics of lesions and rashes. The WHO has warned that the epidemic posed a “substantial danger” to public health worldwide but has refrained from calling it an emergent situation. Healthcare groups, for instance the World Health Network (WHN), meanwhile, indicated a greater level of concern and emphasized the need for swift and united worldwide action against the infection [3].

The zoonotic illness known as monkeypox, which belongs to the genus *Orthopoxviral*, was originally spread from animals to humans. Regarding clinical characteristics, it parallels chickenpox, measles, and smallpox [4]. Ever since the 1970s, monkeypox has been viewed as the orthopoxviral that poses the greatest threat to human well-being. Although it has its inception in Africa, it is frequently observed in metropolitan settings beyond that region [5]. Researchers contend that either variations in lifestyle, or modifications in the basic characteristics of the monkeypox virus, or both, are to blame for the present occurrence of monkeypox in humans on a worldwide scale [6]. Although less extreme, monkeypox shares many of the same patient characteristics as smallpox [7]. As a result, the research and development of third-generation MVA vaccines, such as ACAM3000 and TBC-MVA, is underway [8], and antiviral treatments are still being tested in clinical studies. Eventually, MPX prevention entails limiting contact with infected animals and preventing human-to-human transmission by isolation and observing fundamental hygiene practices until these developments are accessible to individuals residing in isolated endemic regions. It is becoming more and more important to provide health workers with appropriate diagnostic testing, vaccines, and antiviral medications. However, body rashes as well as lesions brought on by a monkeypox illness sometimes mimic those such as chickenpox and cowpox.

The popular polymer chain reaction (PCR) test [9], which is frequently used to diagnose COVID-19 patients [10,11], can be effectively employed to identify the monkeypox viral infection. Recently, COVID-19 diagnosing and seriousness rating using multidimensional medical imaging, such as computed tomography (CT), chest X-rays, and chest ultrasound, has significantly benefited from the use of AI approaches [12,13,14]. The authors of [15] evaluated ten different deep learning (DL) models and attained 99.1% accuracy using a small dataset of 108 patients with COVID-19 and 86 non-COVID-19 patients. The authors of [16] created a method for identifying skin disorders using MobileNet and cellphones. They claimed an accuracy rate of 94.4% in detecting chickenpox symptoms. Few research studies have appeared that indicate the potential use of ML techniques to the diagnosis of monkeypox using image processing techniques. Until the recent appearance of the virus in many countries, there was a dearth of publicly accessible datasets for training and testing purposes, which prevented the development of a framework for the image-based diagnosis of monkeypox. This achievement encourages the research world to use AI methods for diagnosing monkeypox from patient digitized skin scans. It is common knowledge that supervised or semi-supervised AI systems are data-driven and need a lot of data to be developed successfully.

To reduce the spread of the virus within a population, the early identification of monkeypox, matching contact tracing, and immediate isolation are necessary. In this case, automated computer-aided methods based on AI may significantly restrict its global expansion. In the event that enough samples are accessible, DL techniques have been proven to be useful in the automatic classification of skin infections [17,18]. Such deep networks can analyze pictures in various layers, accordingly extracting significant characteristics and acquiring knowledge to select the best approximations for certain tasks when trained with a huge amount of data [19]. The application of DL-based frameworks is constrained by the need for substantial volumes of datasets and time-consuming training using specialized computational capabilities [20]. Transfer learning is also a frequently employed method when data are scarce. CNN-based image classification involves feeding input images into the network, developing a model using DL techniques such as forward and backward propagation, and thereafter classifying new images using the trained model. As a result, the general image classification techniques are support vector machine-based image classification, artificial neural network-based image categorization and CNN-based classification. The k-means clustering technique is suggested as a way to gauge how unique the grouping outcomes are. If the central values stay the same after the clustering technique is applied, the clustering centroids are distinct. It belongs to the unsupervised learning model. In this study, we hypothesize that identifying the real number of clusters will result in more reliable cluster findings. In data mining and image processing applications, clustering is a key approach for aggregating numerical and image data. In the realm of research and development, such as in medical science, clustering is used extensively to group illness symptoms and treatments in order to save time and provide effective outcomes [21]. It is used in marketing, data mining, astronomy, and other fields.

The propagation of the monkeypox virus, its symptoms and indicators, preventative strategies, and protective gear may all be made more widely known thanks to the ready availability of data. In order to battle the present outbreak and improve healthcare services and hygiene standards, it may also be useful as a foundation for studies to better understand the monkeypox virus. Instead of focusing on the past or the future, concentrate on the present moment and embrace what has been occurring worldwide. In this epidemic circumstance, we must expect that we will be worn out and lack motivation, but this is normal. Individuals who are infected by this monkeypox virus need to concentrate on their daily, attainable goals, control expectations, and pay attention to their strengths and accomplishments. In the meanwhile, a fresh approach to determine the value of k in the k-means clustering algorithm was suggested. The image segmentation technique put forth in our study is extensively used and has produced positive outcomes in the area of monkeypox image analysis. Additionally, we investigate the level of healthcare professionals’ satisfaction with LIME’s monkeypox prediction explanations for black-box classification models in this research. The hyperparameters of CNN have been selected using the Bayesian optimization technique. How could one classify the monkeypox virus by using the Bayesian optimization hyperparameter technique? This research will be carried out as part of our future work.

The following is a summary of our key contributions:

- VGG16 [22], VGG19 [22], ResNet50 [23], ResNet101 [23], DenseNet201 [24], and AlexNet [25] are six distinct deep CNN models that have been implemented and evaluated to make the diagnosis with the monkeypox viral infection using skin images;
- We used the k-means clustering technique to improve the accuracy level of monkeypox identification;
- We conducted a thorough examination of the outcomes and compare the performances of the same methods when used on independent datasets of pictures of monkeypox skin;
- Finally, we use LIME to show how the models may predict top characteristics. To support our conclusions, we offer a post-image analysis explanation using LIME.

The rest of the paper is organized as follows. The influence of the monkeypox virus illness is explored in this study. Similar works which were carried out by other researchers are given in Section 2. Section 3 and Section 4 discusses the datasets and model creation, and several matrices and algorithms are also covered. Additionally, Section 5 discusses the evaluation of our proposed model, providing the best results of each of the models employed. In Section 6, the conclusion and its future scope are discussed.

## 2. Related Works

Since the world was impacted by COVID-19 in 2020–2021, the arrival of monkeypox in 2022, as observed by several countries, illustrates additional concern on a global scale. Several experts are currently focusing on this monkeypox virus since its inception in 2022. Several other types of methodologies are being used to forecast, analyze, and categorize this pathogen, including AI, deep learning, machine learning, and re-enforcement learning. COVID-19 was predicted, identified, and classified by many studies using various image processing techniques, such as CT-scan and CXR. The following explains a few literature reviews of COVID-19 and monkeypox virus.

The authors in [26] explore an automated method to accurately classify COVID-19 patients vs. healthy cases using chest CT images. InceptionV3, InceptionResNetV2, Xception, DenseNet121, DenseNet169, and DenseNet201 are some examples of models using pre-trained weights that were looked into. Eventually, they came to the conclusion that DenseNet201 is the best model for COVID-19 detection utilizing a CNN technique and CT characteristics. By using a chest X-ray to identify COVID-19 patients, the authors in [27] created a new, modified classification technique. To deliver the insights, they then used a local LIME. While tweaking a CNN’s transfer learning using the classification approach, hyperparameter values are optimized using the gray wolf optimizer algorithm. Following the classification of a series of X-ray pictures using the trained model, qualitative explanations are carried out. Using a dataset of 842 X-ray pictures, their proposed method performed better than both the baseline transfer learning method and the standard CNN method, with an overall accuracy of 94.76%.

A CNN-tailored Deep Neural Network (DNN) that can jointly train and evaluate both CT scans and CXRs has been developed by the author in [28]. They attained an overall accuracy of 96.28% (AUC = 0.9808 and false negative rate = 0.0208) in their trials. Support vector machines (SVM) based on the AlexNet model are suggested by the author in [29]. Subsequently, via the VGGNet16 technique, the SVM model is created. The suggested methods beat AlexNet and VGG16 DL systems for the classification of chest X-ray images, according to combined deep networks and a strong classifier result. For twelve chest X-ray disorders, the suggested AlexNet and VGGNet-based SVM provides average area under the curve values of 98% and 97%, respectively. Based on the findings of chest computerized tomography (CT) and chest radiographs, the researchers have suggested COVID-19 patient screening in [11] (X-ray). Early investigations have demonstrated a fairly high accuracy in illness diagnosis when combined with AI and DL-based systems for analysis. They apply six alternative Deep Convolutional Neural Networks (DCNN) models—VGG16, MobileNetV2, InceptionResNetV2, ResNet50, ResNet101, and VGG19—and employ a mixed dataset of CT and X-ray images to identify COVID-19 patients in order to further investigate these techniques. A modified MobileNetV2 model outperforms all others, according to preliminary findings, with an accuracy of 95 + 1.12% (AUC = 0.816). A decentralized federated transfer learning approach for collaborative machinery defect diagnosis is suggested in article [30]. For the purpose of streamlining the process of aggregating models, a customized committee consensus scheme is created, and a source data-free transfer learning approach is also put forth. More than 90% testing accuracies may often be attained by implementing the experiments on two decentralized fault diagnostic datasets for validation. In order to solve the issue of sensor malfunction, a DL-based remaining usable life (RUL) prediction approach is put forth in the study [31]. To fully utilize the data from various sensors, a global feature extraction approach is used. In order to derive generalized sensor-invariant characteristics, adversarial learning is also introduced.

Using a state-of-the-art deep DL method, the author of [32] published the open-source “Monkeypox Skin Lesion Dataset (MSLD)” for automatically detecting monkeypox from skin lesions. The author described how the VGG-16, ResNet50, and InceptionV3 pre-trained DL algorithms are used to classify monkeypox and other illnesses. They also created an ensemble of the three approaches. ResNet50 obtains the highest overall accuracy (82.96%), followed by VGG16 (81.48%), and the ensemble system (79.56%). The author of [33] suggested and assessed a modified VGG-16 model. According to their preliminary computational findings, their proposed model can accurately describe patients with monkeypox 97% of the time (AUC = 97.2) and 88% of the time (AUC = 0.867). To further stress that the outcome could be confirmed, physicians next double-checked the claims made by their algorithm. In [34], the author evaluated the viability of diagnosing various forms of pox and measles from digital skin images of lesions and rashes using seven state of-the-art AI classifiers. They created and used a computerized skin dataset that contained images of the skin infections and rashes caused by five distinct illnesses, including cowpox, chickenpox, smallpox, measles, and monkeypox. According to their research, deep implementations offer a significant deal of promise for accurately detecting monkeypox from digital skin pictures (precision of 85%). A large number of training samples are needed to train those deep models in order to obtain a more robust detection capacity. Our research primarily concentrated on using Deep CNN to classify the monkeypox virus. The work completed by the other researchers is displayed in the table below. Finally, Table 1 contains our suggested work with the best desired outcome.

## 3. Methodology

### 3.1. Dataset Description

**Figure 1:**
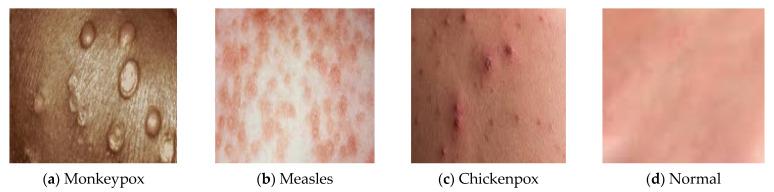
(**a**) depicts the skin images of monkeypox and (**b**–**d**) depict other images such as measles, chickenpox, and normal images, respectively.

Our monkeypox skin image dataset is mostly created from manually searched publicly accessible case reports, news portals, and websites [4]. The classification of “monkeypox” patients from comparable non-monkeypox cases is the major goal of this research. In order to prepare the dataset for binary classification, we also provide skin samples of chickenpox, measles, and normal as the ‘Others’ class. Using Google’s Reverse Image Search and cross-referencing with other sources, all of the skin images were confirmed. All four types of images have been displayed in Figure 1. The dataset used in this study, which contains data on monkeypox, chickenpox, measles and normal images, was acquired from the Kaggle repository. We have gathered 835 samples altogether, from which 432 are classified as “monkeypox”, while the remaining 403 are classified as “others” (i.e., chickenpox, measles and normal). Figure 1 exhibits a few exemplary samples. Reliable information is hard to come by, since the monkeypox outbreak is still in its early stages. In order to prevent patients from being recognized from their matching photographs, we cropped images to remove undesirable background areas and masked the eye region with black boxes. Similar procedures were performed to cover up the exposed intimate areas. In order to prevent excessive stretching of the real skin diseases during image resizing, we included extra blank pixels in the perimeter of many images because conventional AI deep models commonly use square-shaped images as inputs (typically 224 × 224 × 3 pixels). Finally, we used bilinear interpolation to crop and resize each image to 224 × 224 × 3 pixels.

For this work, we used the Monkeypox Image Dataset, which is a publicly available dataset that includes Kaggle images of various body parts (facial, neck, hand, arm, and leg) of patients with monkeypox and non-monkeypox (measles, chickenpox, and normal) instances. There are no datasets that are specifically focused on skin imaging data. Thus, in our classification process, we took into account the skin images from those data. Then, we used the K-means clustering algorithm. We also provide a preliminary feasibility study using transfer learning, DL, and the architectures of VGG16, VGG19, ResNet50, ResNet101, DenseNet201, and AlexNet to investigate the effectiveness of DL algorithms for the early diagnosis of monkeypox virus. Furthermore, we provide LIME, a new explaining approach that learns an interpretable model locally around the prediction to describe the expectations of any classifiers in a true and comprehensible way. LIME is a method that, by using a local approximation with an understandable concept, could faithfully describe the predictions of any classifiers or regression model.

### 3.2. K-Means Clustering

The most prevalent partitioning-based clustering method is the k-means algorithm. It is a clustering approach that is unsupervised. The data points that are comparable to the centroid are allocated to the cluster in which the centroid is located after carefully selecting the centroid and comparing it to the data points based on their intensity and features to determine the distance. Determining the data points closest to the clusters allows for the calculation of new ‘k’ centroids and the formation of new k-clusters. The k-means [10] method can be outlined in the following steps:

➢ Randomly choose k locations and make them the starting centroids.
➢ Choose a data point from the collection, compare it to each centroid, and then place it in the cluster for that centroid if the comparison reveals a match (minimum distance). Ties (equal distance), if any, are broken arbitrarily.
➢ Recalculate the centroid’s values for each k-point clusters once each data point has been allocated to one of the clusters.
➢ Continue the aforementioned procedures until no data point switches from one cluster to another.

### 3.3. Convolutional Neural Network Approach

**Figure 2:**
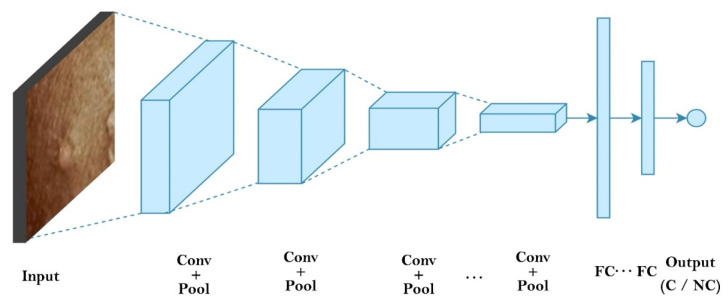
Depicts Convolutional Neural Network model.

**Figure 3:**
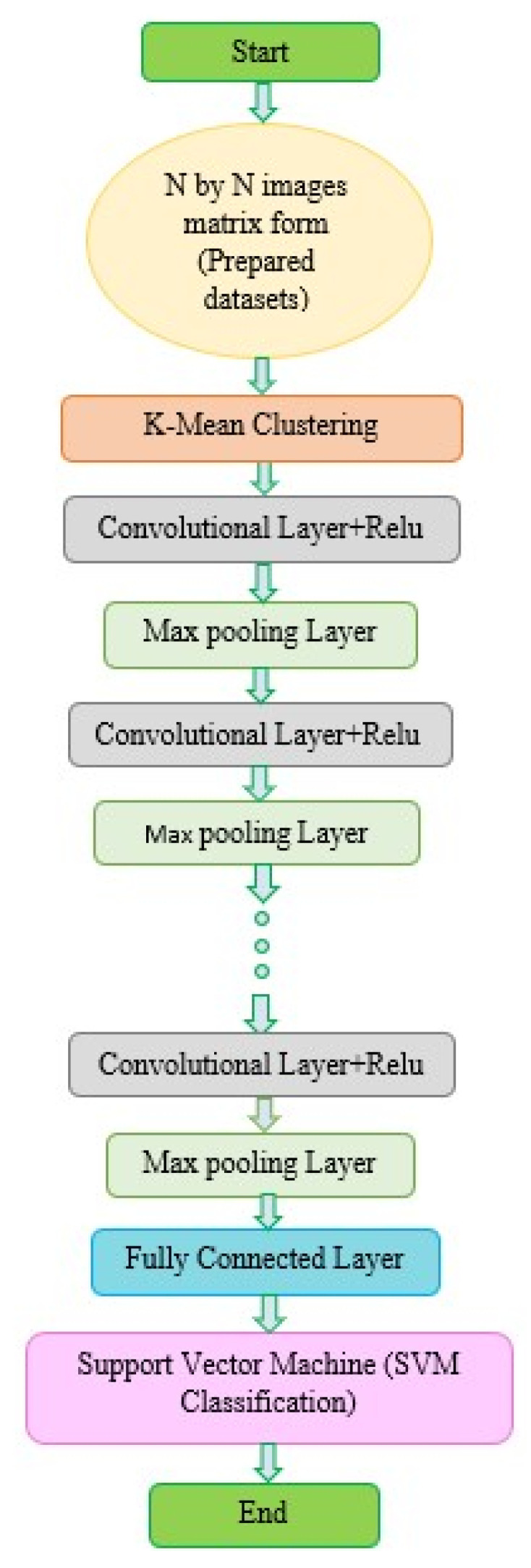
Workflow of our semi-supervised CNN model.

**Figure 4:**
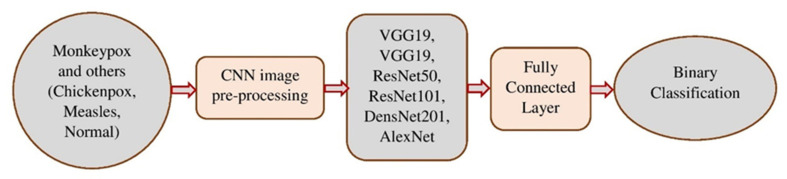
Monkeypox and other binary classification problems using CNN architecture.

The CNN is a well-known DL framework [35]. CNN uses numerous representational layers. With the use of approximation nonlinear functions and nonlinear transformations, CNN can use these major components to extract feature representational characteristics from the source data. A feature extractor made up of many convolutional layers is often proceeded by pooling layers and a SoftMax classifier in a conventional CNN layout. While the pooling layer compresses the dimensions and speeds up processing, the convolutional layer extracts signal characteristics. On its own, this design is capable of achieving some regularization. The best SoftMax is then used to classify the retrieved features. Figure 2 and Figure 3 show our CNN model and the CNN flowchart, respectively. Figure 4 explains the CNN architecture of monkeypox and others for binary classification tasks. The input layer, convolutional layer, pooling layer, fully connected layer, and output layer make up the CNN’s fundamental network model. The specifics of the network components are described as follows.

Input Layer: Image and audio data, among others, may be directly ingested by Deep CNN. However, pre-processing these data is typically required to provide better results.

Convolutional Layer: Utilizing kernels and filters, the convolution layer extracts information and characteristics. The kernel typically scans the input picture’s spatial location point-by-point, and it has a lower size than the input image. Then, bias and other necessary components are added, and the weighted total is determined. The nonlinear activation function is finally applied to the layer’s output to produce fresh features for the following convolutional layer.

Pooling layer: Commonly employed between two convolutional layers is a pooling layer. The characteristics discovered by the preceding convolutional layer are attempted to be compressed. By obtaining the maximum or average value from a certain area, compression is accomplished. Most often, max pooling is utilized, since it produces the greatest results.

Fully Connected Layer: The convolutional and pooling layers give the visual features a place to live. All of the neurons from the layer before are linked to the layer above in a fully connected layer. It may be thought of as a reasonably priced method of learning a linear function from the feature regions.

Output Layer: Depending on the study objective, the Deep Convolutional Neural Network’s output layer completes various tasks. The categorization outcomes are often calculated using the SoftMax algorithm.

This study made use of six pre-trained CNNs: VGG16, VGG19, ResNet50, ResNet101, DenseNet201, and AlexNet.

#### 3.3.1. Classification Model

Deep neural networks such as ResNet50, ResNet101, Densenet201, and AlexNet are utilized to simulate the aspect of monkeypox images contortion, which is then classified by SVM.

In CNN, the SoftMax classifier is frequently employed. The input of the SoftMax layer may be stated as follows, assuming that w is the weight of the penultimate layer to the SoftMax layer, and h is the activation value of the penultimate layer.

(1) $$a_{i}=\sum_{k}h_{k}w_{k}$$

Assume that there are *N* nodes in the SoftMax layer for such an *N*-class classification method, and that every node’s result is registered as $p_{i}$, where i=1,2,…N, and $p_{i}$ is a discrete probability distribution such that $\sum_{i=1}^{N}p_{i}=1$. Among them

(2) $$p_{i}=\frac{\exp(a_{i})}{\sum_{j=1}^{N}a_{j}}$$

The cross-entropy loss function of Softmax is calculated using the solution of Equation (2). CNNs are capable of obtaining visual information; however, they fall short of achieving the best classification performance. The complicated characteristics of the picture cannot be learned by SVM using a fixed kernel function. To acquire any deciding planes, the “soft interval” approach may be utilized to maximize the interval. In the learning feature space, the classifications issue may thus be solved optimally. SVM is frequently employed in data analysis, pattern identification, regression analysis (SVR), as well as other areas such as a standard supervised machine learning technique. Standard SVM is a non-probabilistic binary linear classifier; that is, for each input, it predicts that the input belongs to one of the two categories [36]. The basic principle of SVM [37] is as follows.

Set up the training set data samples as follows: $\{(x_{i},y_{i})|x_{i}\in R^{d},y_{i}\in \{-1,1\},i=1,2,\ldots ,N\},\;y_{i}$ for the category name, N for the training datasets, and d for the data’s dimension. There is a generalized optimum categorization hyperplane for linearly separable data sets:

(3) w.x+b=0

These factors combine to give the classifications interval the optimum output, with $\frac{2}{\left\|w\right\|}$ being the greatest and $\frac{1}{2}{\left\|w\right\|}^{2}$ being the lowest. Amongst those, w is a n-dimensional vector, b is an offset, and dot is an inner product operator. As a consequence, optimizing issue categorization may be changed into the following types:

(4) $$\min\frac{1}{2}{\left\|w\right\|}^{2}such\;that\;y_{i}(w^{T}x_{i}+b)\geq 1,\;i=1,2,\ldots ,n$$

Based on empirical risk reduction, CNN’s learning algorithm works to reduce training sample errors. Regardless of whether it is local or global optimum, the training procedure will end when the first classification hyperplane is discovered using the backpropagation method. The structural risk reduction principle is used to classify SVM in the most advantageous way possible universally. As may be observed, multilayer neural networks have less generalization potential than SVM. As a result, replacing CNN’s SoftMax layer with SVM will improve classification performance. In Figure 3 and Figure 4, the two classification problems are displayed. The classification assessments: 20% of the data was saved for testing, while the remaining 80% was used for training.

#### 3.3.2. Pre-Trained Models

The VGG16, VGG19, ResNet50, ResNet101, DenseNet201, and AlexNet models were six of the pre-trained CNNs that we employed. We applied the image resizing to a standardized target size of 224 × 224 pixels, which would be consistent with the default input size of the chosen VGG16, VGG19, ResNet50, ResNet101, DenseNet201, and AlexNet CNN architectures. This is because the images in the extended COVID-19 image data collection are in a variety of sizes. Furthermore, to avoid over-fitting, which frequently happens when working with pre-trained sophisticated CNN models and limited samples, we utilized an image augmentation approach during the training phase.

#### 3.3.3. VGG16 and VGG19

**Figure 5:**
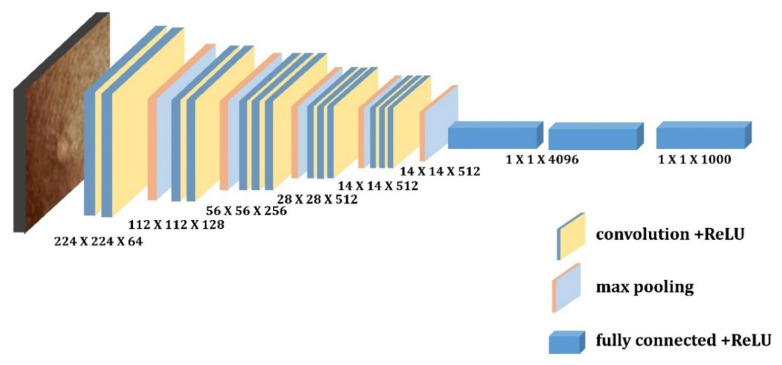
Depicts architecture of VGG-16.

**Figure 6:**
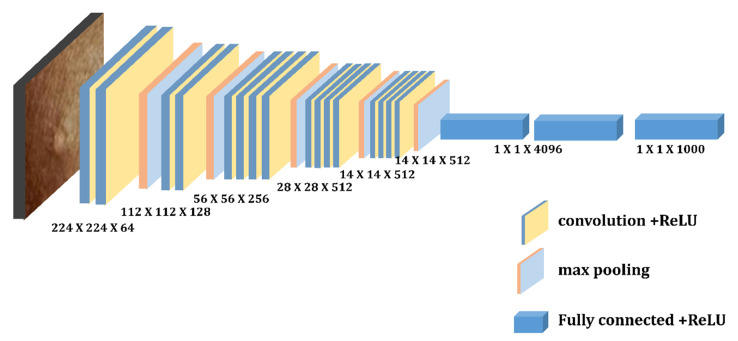
Depicts architecture of VGG-19.

Simonyan and Zisserman first presented VGG designs in 2014 [38]. This network made use of 3 × 3 convolutional layers that were further separated from one another. VGG16 and VGG19 are two different VGG designs, where 16 and 19, respectively, represent the number of weight layers in the network. For instance, VGG-16 has 13 convolutional layers, 2 fully connected layers and 1 SoftMax classifier, and VGG19 is a convolutional neural network that is 19 layers deep, including 16 convolution layers, 3 fully connected layers, 5 max pooling layers and 1 soft-max layer. The RGB-channel images in the ImageNet dataset have a fixed size of 224 × 224 [22]. Figure 5 and Figure 6 depict the architecture of these two pretrained models, respectively.

#### 3.3.4. ResNet50 and ResNet101

**Figure 7:**
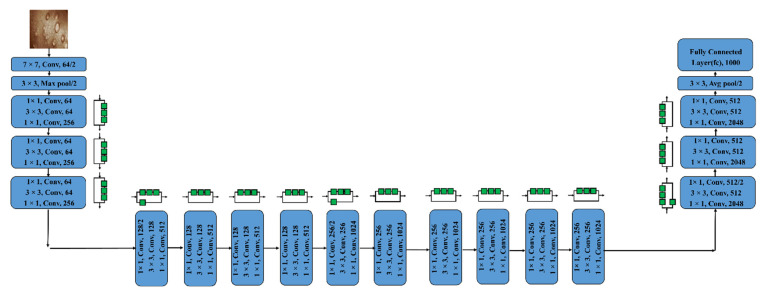
Depicts architecture of ResNet50.

**Figure 8:**
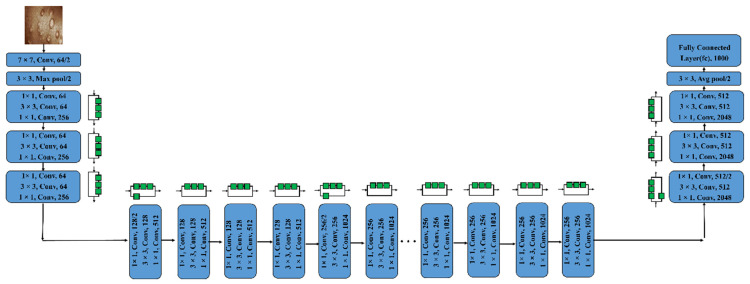
Depicts architecture of ResNet101.

The idea of residual blocks served as the foundation for the ResNet model’s creation. It is a specific type of CNN that Kaimimg established in 2015 [39]. Convolution procedures are followed by Batch Normalization and ReLU nonlinearity in the residue modules of this structure. The inputs can forward-propagate very quickly as well as extract features very effectively thanks to these blocks. Residual Neural Network with 50 deep layers is known as Resnet-50. A CNN with 101 layers is identified as ResNet-101. We may utilize the ImageNet databases to populate the network’s pre-trained model, as that network has been trained on more than one million images. The 224 × 224-pixel image is the input size for the network [23]. Figure 7 and Figure 8 depict the framework of these two pre-trained models, respectively.

#### 3.3.5. DenseNet201 and AlexNet

**Figure 9:**
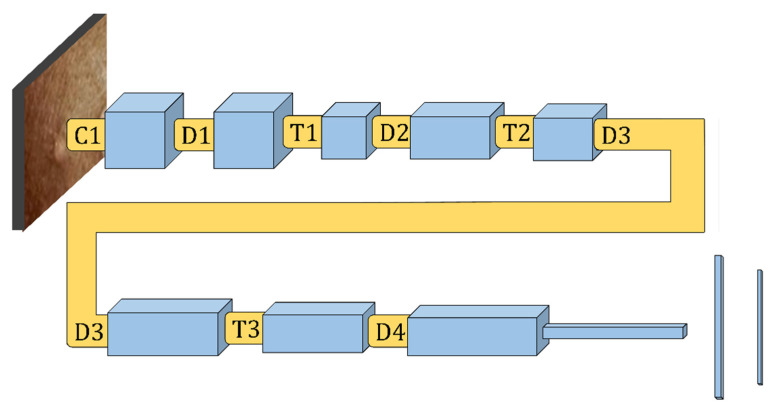
Depicts architecture of DenseNet201.

DenseNet201, an NN with 201 layers, is one of the NNs for visual object recognition. The input image size for the network is 224 × 224 pixels. The vanishing gradient problem in deep networks caused accuracy to drop, and DenseNet was created to address this issue. Every layer accepts as an input the output feature maps from all the preceding layers since the layers are coupled together in dense blocks. The smaller interconnections in this design enable each layer to receive more supervision from the loss function. Levels linked to each of the layers before them make a thick block. The input’s spatial dimension is reduced by a transition layer [24]. Figure 9 shows the DenseNet201 design.

**Figure 10:**
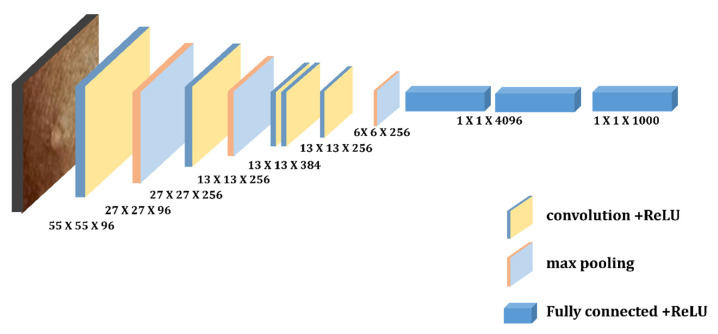
Depicts architecture of AlexNet.

Krizhevsky et al. [40] suggested the AlexNet framework. It is an eight-layer NN with three full connection layers, three pooling layers, and five convolutional layers. The input photos are used as the first convolution layer, which resizes each image to 224 × 224 using 96 kernels. The operation was then carried on. To increase the accuracy and speed in Alexnet, the authors employed the ReLU activation function and Dropout. The AlexNet model’s structure [25] is depicted in Figure 10.

#### 3.3.6. Model Evaluation

**Figure 11:**
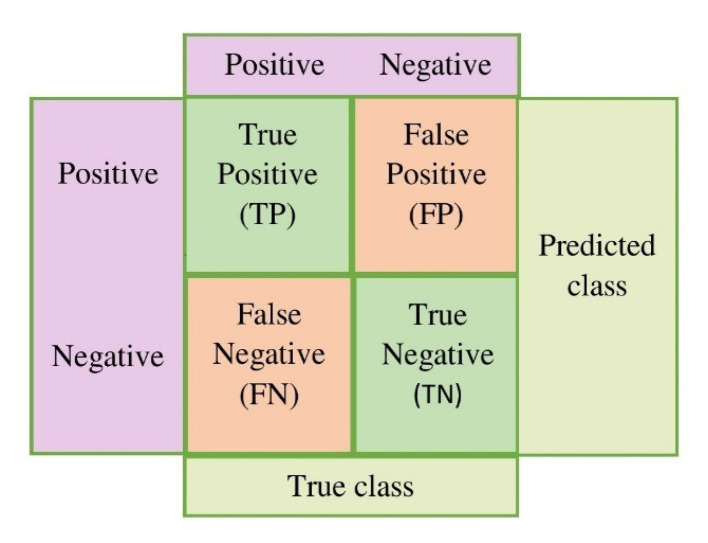
Confusion Matrix.

We discuss the assessment measures used to confirm the effectiveness of the suggested technique in this section. Typically, accuracy is used to describe the classification results. However, in medical imaging, model fidelity is not sufficient to have a precise understanding of the model. Therefore, there are several additional metrics, such as accuracy, precision, recall, ROC curve, and F1-score, to assess a DL model, whereas the ROC AUC value aids in comprehending the separable capacity of a certain classifier. The general form of the confusion matrix is shown in Figure 11.

All of these indicators have been utilized by us to assess and comprehend a model’s performance. A confusion matrix, which is based on the following, is seen to be the most thorough approach to describe all the measurements.

True Positive (TP): patients with monkeypox infection are categorized as patients;

False Positive (FP): others identified as having the monkey pox;

True Negative (TN): classifying others as others;

False Negative (FN): Patients with monkeypox infection were categorized as others.

The patients affected by chickenpox, measles, and normal are represented by others. Therefore, a targeting indication that has been properly categorized should be True Positive (TP) or True Negative (TN), which is similar to how incorrect target labeling classification results in False Positive (FP) or False Negative (FN) results (FN). Accuracy, precision, recall, and F1-score values are computed using the following formulas.

Precision: The ratio of correctly predicted positive events to all expected positive outcomes is used to measure precision.

$$\mathrm{Pr}ecision=\frac{\sum TP}{\sum TP+\sum FP}$$

Sensitivity: The only precise positive metric that is proportional to the total number of occurrences is called sensitivity and may be calculated as follows:

$$Sensitivity=\frac{\sum TP}{\sum TP+\sum FN}$$

Recall and “True Positive Rate” are two more terms for sensitivity (TPR)

Specificity: The number of correctly detected and computed true negatives is known as specificity, and it may be determined using the method below.

$$Specificity=\frac{\sum TN}{\sum TN+\sum FP}$$

Accuracy: The total number of occurrences that were correctly recognized throughout all cases is the accuracy. Accuracy can be assessed by

$$Accuracy=\frac{\sum TP+\sum TN}{\sum TP+\sum FP+\sum FN+\sum TN}$$

F1-score: The harmonic mean of recall and precision is known as the F1-score. The highest possible F1-score is 1, which denotes flawless precision and recall.

F1−Score=2×Recall×PrecisionRecall+Precision

Area Under Curve (AUC): The AUC depicts how the models behave under various circumstances. It is calculable as

$$AreaUnder\;Curve=\frac{Sensitivity}{2}+\frac{Specificity}{2}$$

## 4. Local Interpretable Model-Agnostic Explanations (LIME)

Here, we discuss LIME, whose main objective is to find an interpretable model that is locally accurate to the classifier across the interpretable representations. LIME was used to describe the CNNs’ categorization [41]. It is a technique for training a straightforward, understandable linear model to mimic any black box model’s decision rule, including a CNN. In other words, it is a method that enables comprehension of the input properties of the DL models which impact its predictions, and it is used to interpret the overall prediction. LIME has received much attention recently due to its outstanding performance in explaining the intricacies of picture categorization [42]. For image classifiers, LIME starts by constructing super-pixels, which are groups of pixels that have similar attributes such as pixel intensity. These super-pixels serve as an interpretable representation of the input pictures. LIME samples data comparable to the main instance and generates predictions for them using the original black box model to explain the provided instance. Afterwards, an interpretable linear model (explainer) is fitted to the sampled instances and predictions as a new training dataset. The portions of an image that are important to a certain prediction are subsequently shown on saliency maps created by the explainer. Here, LIME was used to identify expected traits in order to comprehend how the systems made decisions. The LIME results will be discussed in the following section.

## 5. Results and Discussion

We used MATLAB software to implement the tools mentioned above. The Lenovo Intel(R) Core (TM)i5-10210U CPU @1.60 GHz 2.11 GHz system is used for all experiments. It has the following specifications: 8.00 GB Random Access Memory (RAM), 512 GB Solid-State Drive. Classification of the monkeypox virus using deep CNN, which is more accurate than the existing state-of-the-art approaches, is the main objective of this research. The following sub-sections discuss how the classification model works. We have provided the output results for our proposed model.

### 5.1. K-Means Clustering Results

**Figure 12:**
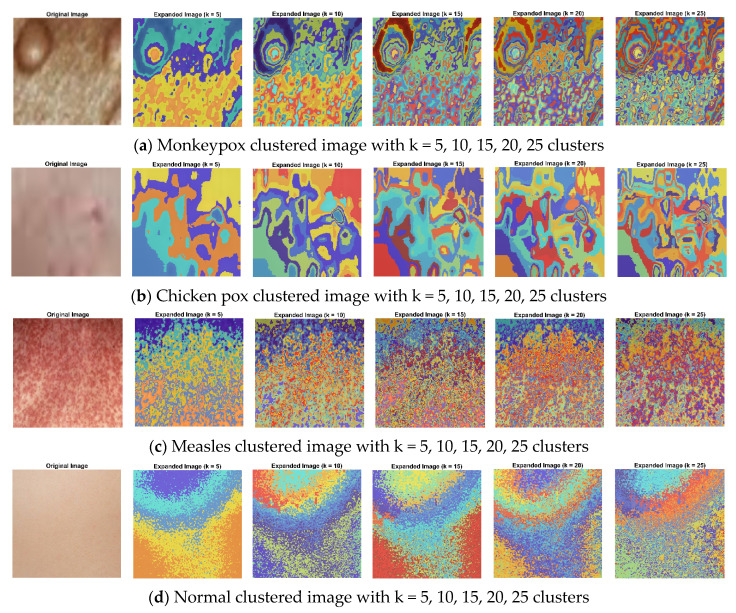
(**a**–**d**) represents k-means clustering images with k = 5, 10, 15, 20, 25 clusters of monkeypox, chickenpox, measles, and normal images, respectively.

A technique to divide groups of items into homogeneous sub-groups is called data clustering. Each data item is treated as having a position in Euclidean space when using the k-means clustering. It locates divisions so items in each cluster are as close to one another and as far away from one another as feasible. The “imsegkmeans” function may be used to group picture pixels inside a color space according to value. The following graphic demonstrates how utilizing multiple color spaces might enhance segmentation results by performing k-means clustering on an image in various color spaces. Throughout the experiment, the k value was fixed for each method and varied as 5, 10, 15, 20, and 25 for all the datasets. As a result, for k = 20, uniqueness has been taken into account as the actual value of k. Because the closest clusters are constantly joined with one another, uniqueness can be attained for fewer groups than the real value of k. Figure 12 explicates k-means clustering of images with k = 5, 10, 15, 20, 25 clusters, where (a) is the monkeypox clustering image with k = 5, 10, 15, 20, 25 clusters and (b–d) represent the k-means clustering images of other (chicken pox, measles, normal) types of skin images.

### 5.2. Results of Monkeypox vs. Others (Chickenpox, Measles and Normal) Pre-Trained Classification Model

We describe our preliminary results for the identification of monkeypox skin using deep CNN models in this paper. Whereas the resulting classification performance is highly encouraging, various limitations prevent the findings from being used more widely. This research creates an ensemble model that combines CNN and SVM in order to enhance the classification accuracy. The tests to classify skin images are conducted to validate the methods classification impact and confirm that the suggested model has a superior performance. We analyzed images of monkeypox and other portions of images containing chickenpox, measles, and normal images to evaluate the accuracy of the pre-trained models we had chosen for binary classification. Table 2, Table 3, Table 4, Table 5, Table 6 and Table 7 provide a summary of the results along with the precision, recall, specificity, accuracy, F1-score, and AUC. ResNet101 produces the greatest accuracy of 94.25%. We compare the classification performance of our six state-of-the-art deep CNN models in this section, as shown in last table (Section 5.2).

For the patient to receive timely medical attention and to lessen the risk of disease transmission, early diagnosis is essential. For this, skin images gathered from patients with the virus had been used. The transfer learning method is used to classify those images. It could be useful in clinical practice, since the classifier performance calculation has a high accuracy rate of 94.25%. Table 2, Table 3, Table 4, Table 5, Table 6 and Table 7 provide a summary of the performance matrices for the various CNN algorithms examined for each of the six different categorization techniques. Table 2 shows the performance categorization of the VGG-16 model, which has an accuracy of 92.57% with an AUC of 98.11% and a loss of 0.1005. With an AUC of 96.94% and a loss of 0.1411, the VGG-19 model’s accuracy is 90.89%, according to Table 3. The ResNet50 model’s performance is categorized in Table 4 with an AUC of 98.46%, a loss of 0.0813, and an accuracy of 94.05%.

The ResNet101 model’s performance is categorized in Table 5 with an AUC of 98.59%, a loss of 0.0550, and an accuracy of 94.25%. Table 6 shows the performance categorization of the DenseNet201 model, which has an accuracy of 94.05% with an AUC of 98.35% and a loss of 0.0789. With an AUC of 94.39% and a loss of 0.1364, the AlexNet model’s accuracy is 87.53%, according to Table 7. In terms of several evaluation metrics, VGG-16 outperforms other models in five distinct classifying techniques. Table 8 shows the quantitative comparison of the ensemble approach’s 5-fold cross-validation estimates of the ensemble’s mean precision, mean recall, mean F1-score, and mean accuracy for all classes. Table 9 represents the comparison of state-of-the-art methods. This chart illustrates that when compared to the results of individual deep model summaries, the ensemble technique performs better across the board, especially in terms of accuracy (94%). The results are shown with confidence intervals (CI) of 95% in order to give an appropriate overview of the statical significance because the dataset only included a limited number of data items. Despite having such a small dataset, performance of the model as a whole could still be deemed adequate.

**Figure 13:**
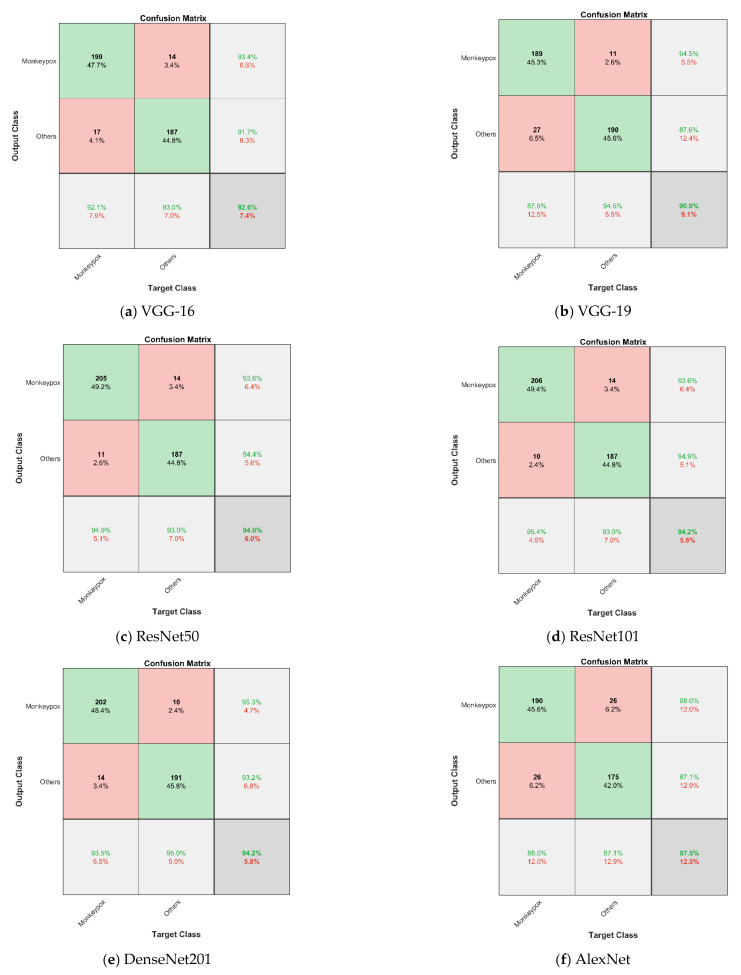
(**a**–**f**) Depicts the confusion matrices of monkeypox virus and other images for VGG-16, VGG-19, ResNet50, ResNet101, DenseNet201 and AlexNet, respectively.

Table 5 contrasts how well the ResNet101 performed. Regarding accuracy, the ResNet101 model performs best in terms of sensitivity, specificity, precision, F1-score, and accuracy. In consideration of this, the ResNet101-based model outperforms all other backbone-based testing approaches. We provide the binary classification confusion matrices for the VGG16, VGG19, ResNet50, ResNet101, DenseNet201, and AlexNet models in Figure 13.

**Figure 14:**
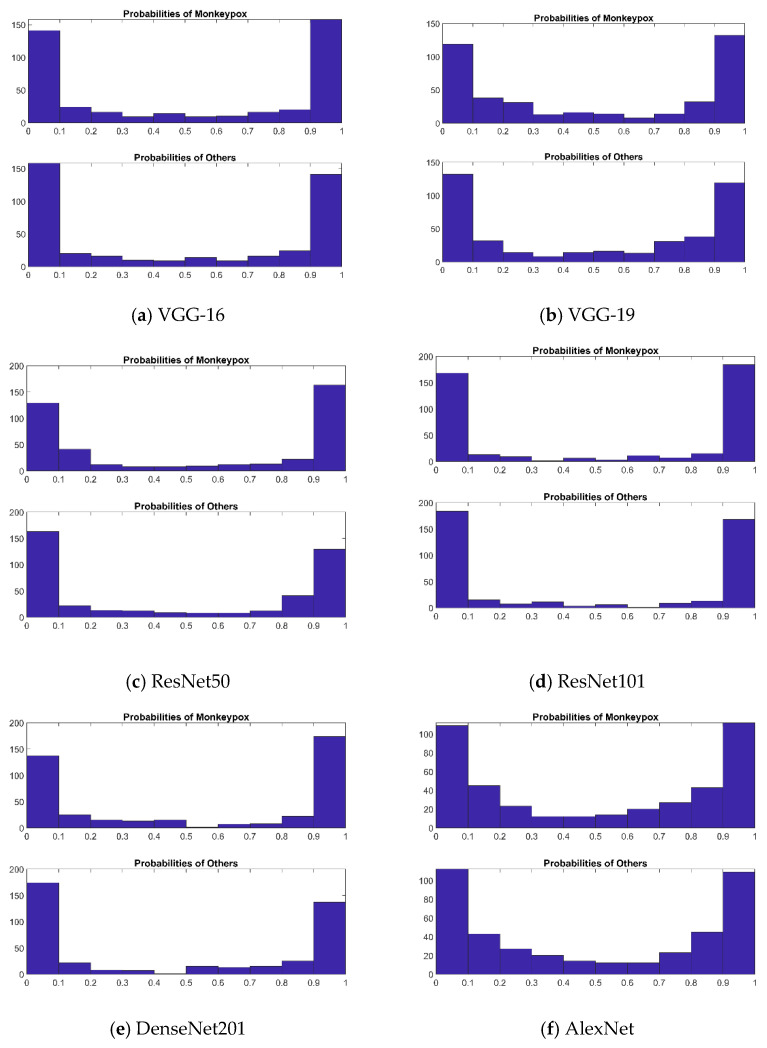
(**a**–**f**) Depicts the predicted probability scores of monkeypox virus and other images by VGG-16, VGG-19, ResNet50, ResNet101, DenseNet201 and AlexNet, respectively.

Every image should be assigned a probability by these algorithms which shows how probable it is to be classified as monkeypox as we concentrated on our six well-known pre-trained CNN models. The binary label indicating whether or not the image is of monkeypox may be produced by comparing these probabilities with a cut-off threshold. The probabilities are displayed in Figure 14. An ideal result ought to be able to forecast the probability of all monkeypox samples being close to 1 and all other samples (chickenpox, measles, and normal) being close to 0. Using these probabilities, we could rapidly establish which sickness group a patient falls into. When compared to the other methods, ResNet101 has the best predicting outcomes for probability. It is straightforward to determine which illness group a patient falls under using this probability distribution. ResNet101 outperformed all other models in terms of prediction probability.

**Figure 15:**
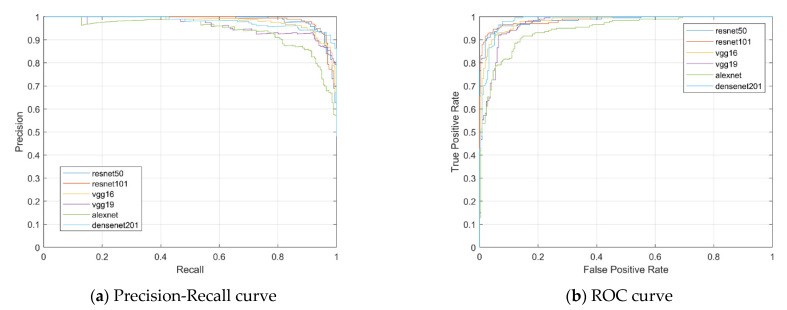
(**a**,**b**) representing precision–recall curve and ROC curve, respectively.

To summarize the results of each of these approaches, we have provided the ROC curves. We conducted a complete exploratory analysis of the achievements of various methods with respect to precision, sensitivity, specificity, accuracy, F1-score, ROC AUC curve, precision–recall curve, and a histogram of the probability model. The recommended techniques outperform current techniques for categorizing the monkeypox virus and other skin conditions. The precision–recall curves for the test set are shown in Figure 15a for the six CNN models. Figure 15b shows measurements of the ROC AUC with the test set’s True Positive Rate (TPR) and False Positive Rate (FPR) shown on the vertical axis and horizontal axis, respectively. Figure 15b compares the ROC curves for monkeypox versus other diseases for six CNN architectures. The ROC curve is produced by plotting the FPR vs. the TPR. This shows that the ROC curves of these six models perform similarly. The best performance is displayed by ResNet101 (AUC = 0.9859). According to Table 1, the best performance was noted when it received an AUC score of 0.9856.

Additionally, investigating the suggested frameworks with LIME demonstrated how ResNet101 helped categorize monkeypox by spotting crucial details in skin images. Here, LIME was used to identify expected traits in order to comprehend how the models made decisions. In this research, we use LIME to explain the results of four widely used pre-trained ImageNet CNNs.

### 5.3. Interpretable Representation of Model Results

Evaluating faith and confidence in predicted output is among the most significant challenges while using any type of classification algorithm for decision makers. This is particularly true when such concepts are employed as a mission-critical component in implementations or are used in fields such as medicine, where predictions cannot be operated upon blindly because the consequences might well be disastrous [32]. The models are frequently assessed using a variety of metrics and a test dataset that is readily accessible, but the metrics may not always be representative of the models’ objectives. As a result, examining specific instances and their interpretable representation is a suitable supplementary strategy to assist us to better appreciate and have confidence in our prediction model while also providing us with important insights into how our model interprets the data.

We developed a LIME object using a decision tree basic model and trained the classification model. When we create a LIME object, we need to specify a query point and the number of important predictors so that the software generates samples of a synthetic dataset and fits a simple model for the query point with important predictors. Then, using the object function plot, we depict the predictor significance in the basic model. Monkeypox, chickenpox, measles, and normal are all included in the data collection.

**Figure 16:**
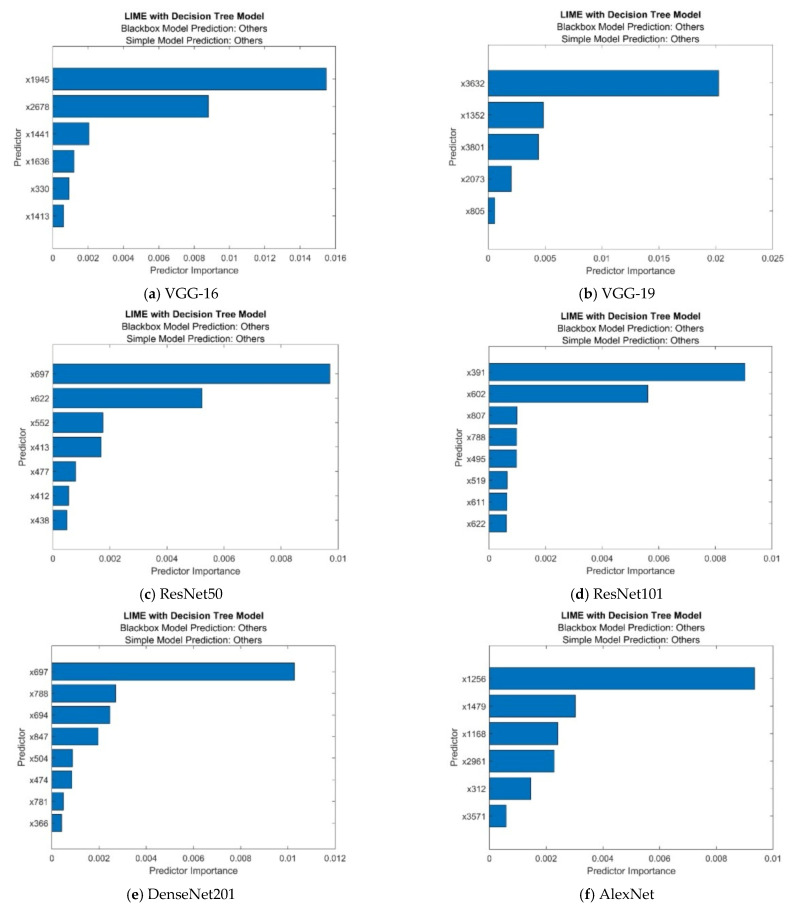
Explanations produced with LIME.

Figure 16 displays the outcomes of the LIME object plotted using an object function graph. We obtained 7 to 10 estimates, which correspond to the blackbox fitted characteristic and the simple model fitted characteristic of the findings, as shown on the question spot. The sorted prediction significance values are displayed in a horizontal bar diagram. Using data tips or bar attributes, we could determine the bar lengths. One of the top models, ResNet101 LIME, has the best MATLAB results, as seen in Table 10.

**Figure 17:**
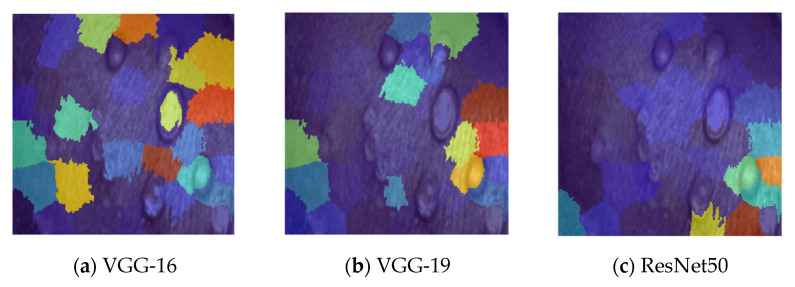

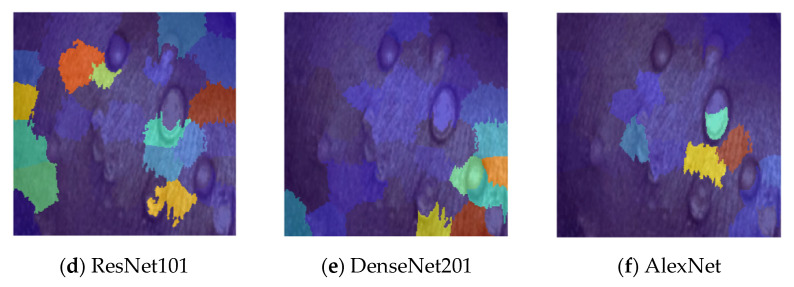
(**a**–**f**) Examples of LIME-based prediction model outcomes that are clearly explained. Images with confirmed monkeypox conditions are shown above for all pre-trained models.

To see which areas of an image are crucial to a network’s categorization decision, we have used the image LIME tool. We have taken all out pre-trained network into consideration in Figure 17. Initially, we had to import the image and scale it down to fit the network’s input size. The image was therefore classified in order to receive a classification model. Following the computation made for the feature importance map, the feature map was also acquired. We chose the 64-feature count, the image segmentation algorithm, and the 5000 artificial samples. To examine which parts of the image have an impact on the categorization score, we plot the results with transparency over the actual image. Figure 17 presents a few test examples of the data that were employed together with the matching interpretable representations of our top-performing predictive pre-trained model. By using the LIME approach, interpretable representations are created, with the shades belonging to green groups of pixels (super-pixels) indicating identification portions of the image that have a favorable impact on a particular target domain and the shades belonging to red super-pixels indicating those portions that have a negative impact. In other ways, the green super-pixels in images labeled (a) have a positive influence on the classification of images of the monkeypox, whereas the green super-pixels in images labeled (b) have a positive impact on the classification of images as no other pathology discovered. By setup, image LIME segments the input sequence into super pixels in order to identify characteristics in the images. Here, we segmented the image into individual features using the “Segmentation” option. The image displays whichever portions of the image are more significant for the classification of the region.

Once the system assesses the net class score for the class indicated by the labels, it uses the LIME approach to create a mapping of the significance of the features in the source image. This feature is employed to justify categorization choices and confirm that our network is concentrating on the relevant image characteristics. By employing a more straightforward, understandable model, this method simulates the categorization performance of the net. The image LIME algorithm analyzes the significance of each input parameter to the network’s identification scoring system for the classes indicated by labels by producing new data from source, classifying the simulated data using nets, and then using the findings to create a straightforward regression model. Machine learning and statistics are required for this task. The LIME explanation result agrees with the system’s statistical analysis as provided by us. It makes clear the significance of every characteristic, how it interacts with certain other characteristics, and how it relates to the classes.

The CNN’s hyperparameters were not chosen using Bayesian optimization in our work. A surrogate model will be used in Bayesian optimization and is adapted to the data of the real model. A complete training of the underlying CNN model using hyperparameters selected especially for that observation constitutes one observation in our context. For each iteration, a set of hyperparameters is chosen, and an observation is made after that. The observation is evaluated using the validation accuracy. Using an acquisition function that balances the options of investigating the whole search universe and taking use of the search space’s high-performing regions, the hyperparameter set is chosen. Future plans call for the implementation of Bayesian optimization, which is a sophisticated method for generating optimal hyperparameters. These are all a few limitations in our work. If we carried out this for our upcoming works, then hyperparameters can be determined by using this for obtaining better results.

## 6. Conclusions

Employing six pre-trained Deep CNNs, our article examined the effectiveness and interpretability of transfer learning. In this study, Deep CNN is used to classify the monkeypox virus and other skin images (chickenpox, measles, normal). K-means clustering is used for the segmentation. For the purpose of identifying images of monkeypox, we have examined the models employing pre-trained weights, also known as transfer learning, including VGG16, VGG19, ResNet50, ResNet101, DenseNet201, and AlexNet. The acquired findings demonstrated the model’s great results, with ResNet101 attaining 94.25% accuracy with 98.59 AUC. Thereafter, we employed the LIME to offer the correct justification for the values predicted by our model. We implemented a LIME to give insights into the monkeypox virus relying on the categorization of different skin images after being motivated by the model’s predicted performance. We are optimistic that this dataset could lead to new research directions for the development of remotely deployable computer-aided diagnostic tools for widespread assessment and early monkeypox identification, particularly in situations where conventional testing techniques are not accessible. We furthermore think our pre-trained and LIME modeling will let monkeypox suspects perform preliminary screening from the comfort of their homes and empower them with the capability to respond appropriately in the initial stages of the illness. One of the most important parts of the medical world is the categorization of the monkeypox virus. It is difficult to create an effective CNN. This necessitates the use of optimization techniques to set CNN hyperparameters absolutely helpful. In the future, we will use a new system that divides categorization into four groups. The Bayesian optimization approach will be used to choose the model’s ideal hyperparameter values.

## Figures and Tables

**Table 1:** Comparison of the suggested strategy against the current one to validate it.

Authors	Image Types	Method Used	Best Accuracy
Cuong D, et al. [26]	Chest CT images	CNN (VGG16, VGG19, InceptionV3, InceptionResNetV2, Xception, DenseNet121, DenseNet169, and DenseNet201)	85%
Grega Vrbančič, et al. [27]	Chest X-ray images	CNN (VGG-19)	94.76%
Himadri, et al. [28]	CT scans and chest X-rays	CNN, DCNN (InceptionV3, MobileNet, and ResNet)	96.28%
Khaled Almezhghwi, et al. [29]	Chest X-ray images	SVM based VGGNet, AlexNet	98%
Md Manjurul Ahsan, et al. [11]	CT scans and chest X-rays	DCNN (VGG16, MobileNetV2, InceptionResNetV2, ResNet50, ResNet101, and VGG19)	98.5%
S N Ali, et al. [32]	Monkeypox, chickenpox, measles	Deep Learning (VGG-16, ResNet50, Inception V3)	82.96%
Ahsan MM, et al. [33]	Monkeypox, chickenpox, measles, normal	Machine Learning (Modified VGG-16)	97%
T Islam, et al. [34]	Monkeypox, chickenpox, smallpox, cowpox, measles, healthy	Artificial Intelligence (ResNet50, Inception-V3, DenseNet121, MnasNet-A1, MobileNet-V2, ShuffleNet-V2, SqueezeNet)	79%
**Proposed work**	**Monkeypox, chickenpox, measles, normal**	**Deep Convolutional Neural Network (VGG-16, VGG-19, ResNet50, ResNet101, DenseNet201, AlexNet)**	**94.25%**

**Table 2:** Performance of VGG-16.

Name	Classes		MacroAVG	MicroAVG
True Positive	199	187	193	193
False Positive	14	17	15.5	15.5
False Negative	17	14	15.5	15.5
True Negative	187	199	193	193
Precision	0.93427	0.91667	0.92547	0.92566
Sensitivity	0.9213	0.93035	0.92582	0.92566
Specificity	0.93035	0.9213	0.92582	0.92566
Accuracy	0.92566	0.92566	0.92566	0.92566
F-measure	0.92774	0.92346	0.9256	0.92566
AUC	0.9811			
Loss	0.1005			

**Table 3:** Performance of VGG-19.

Name	Classes		MacroAVG	MicroAVG
True Positive	189	190	189.5	189.5
False Positive	11	27	19	19
False Negative	27	11	19	19
True Negative	190	189	189.5	189.5
Precision	0.945	0.87558	0.91029	0.90887
Sensitivity	0.825	0.94527	0.91014	0.90887
Specificity	0.94527	0.875	0.91014	0.90887
Accuracy	0.90887	0.90887	0.90887	0.90887
F-measure	0.90865	0.90909	0.90887	0.90887
AUC	0.9694			
Loss	0.1411			

**Table 4:** Performance of ResNet50.

Name	Classes		MacroAVG	MicroAVG
True Positive	205	187	196	196
False Positive	14	11	12.5	12.5
False Negative	11	14	12.5	12.5
True Negative	187	205	196	196
Precision	0.93607	0.94444	0.94026	0.94005
Sensitivity	0.94907	0.93035	0.93971	0.94005
Specificity	0.93035	0.94907	0.93971	0.94005
Accuracy	0.94005	0.94005	0.94005	0.94005
F-measure	0.94253	0.93734	0.93994	0.94005
AUC	0.9846			
Loss	0.0813			

**Table 5:** Performance of ResNet101.

Name	Classes		MacroAVG	MicroAVG
True Positive	206	187	196.5	196.5
False Positive	14	10	12	12
False Negative	10	14	12	12
True Negative	187	206	196.5	196.5
Precision	0.93636	0.94924	0.9428	0.94245
Sensitivity	0.9537	0.93035	0.94203	0.94245
Specificity	0.93035	0.9537	0.94203	0.94245
Accuracy	**0.94495**	**0.94245**	**0.94245**	**0.94245**
F-measure	0.94495	0.9397	0.94233	0.94245
AUC	0.9859			
Loss	0.0550			

**Table 6:** Performance of DenseNet201.

Name	Classes		MacroAVG	MicroAVG
True Positive	202	191	196.5	196.5
False Positive	10	14	12	12
False Negative	14	10	12	12
True Negative	191	202	196.5	196.5
Precision	0.95283	0.93171	0.94227	0.94245
Sensitivity	0.93519	0.95025	0.94227	0.94145
Specificity	0.95025	0.93519	0.94227	0.94045
Accuracy	0.94245	0.94245	0.94045	0.94045
F-measure	0.94393	0.94089	0.94241	0.94245
AUC	0.9835			
Loss	0.0789			

**Table 7:** Performance of AlexNet.

Name	Classes		MacroAVG	MicroAVG
True Positive	190	175	182.5	182.5
False Positive	26	26	26	26
False Negative	26	26	26	26
True Negative	175	190	182.5	182.5
Precision	0.87963	0.87065	0.87514	0.8753
Sensitivity	0.87963	0.87065	0.87514	0.8753
Specificity	0.87065	0.87963	0.87514	0.8753
Accuracy	0.8753	0.8753	0.8753	0.8753
F-measure	0.87963	0.87065	0.87514	0.8753
AUC	0.9439			
Loss	0.1364			

**Table 8:** The comparison of mean precision, mean sensitivity, mean specificity, mean accuracy and mean F-score over the 5-fold cross-validation.

Models	Mean Precision	Mean Sensitivity	Mean Specificity	Mean Accuracy	F-Score
VGG-16	0.92	0.92	0.92	0.92	0.92
VGG-19	0.90	0.90	0.90	0.90	0.90
ResNet50	0.94	0.94	0.94	0.94	0.94
ResNet101	0.94	0.94	0.94	0.94	0.94
DenseNet 201	0.94	0.94	0.94	0.94	0.94
AlexNet	0.87	0.87	0.87	0.87	0.87

**Table 9:** Performance comparison of state-of-the-art method.

Models	Precision	Sensitivity	Specificity	Accuracy	F-score	AUC	Loss
VGG-16	92%	92%	92%	92%	92%	98%	10%
VGG-19	91%	91%	91%	91%	91%	96%	14%
ResNet50	94%	94%	94%	94%	94%	98%	08%
ResNet101	**94%**	**94%**	**94%**	**94%**	**94%**	**98%**	**05%**
DenseNet 201	94%	94%	94%	94%	94%	98%	07%
AlexNet	87%	87%	87%	87%	87%	94%	13%

**Table 10:** Lime result for ResNet 101 model.

Lime Result = lime with properties	: (ResNet 101)
BlackboxModel	: [1 × 1 ClassificationECOC]
DataLocality	: ‘global’
CategoricalPredictors	: []
Type	: ‘classification’
X	: [418 × 4096 double]
QueryPoint	: [−5.3655…]
NumImportantPredictors	: 10
NumSyntheticData	: 5000
SyntheticData	: [5000 × 1000 double]
Fitted	: {5000 × 1 cell}
SimpleModel	: [1 × 1 ClassificationTree]
ImportantPredictors	: [8 × 1 double]
BlackboxFitted	: {‘Others’}
SimpleModelFitted	: {‘Others’}

## Data Availability

The data used in this paper is available in the references in Section 3.1.
